# From waste to surface modification of aluminum bronze using selective surface diffusion process

**DOI:** 10.1038/s41598-018-38120-2

**Published:** 2019-02-07

**Authors:** Isha Singla, Himanish Kumar, Farshid Pahlevani, Wilson Handoko, Sagar T. Cholake, Rumana Hossain, Veena Sahajwalla

**Affiliations:** 1 Centre for Sustainable Materials Research and Technology (SMART), School of Materials Science and Engineering, UNSW Sydney, Sydney, NSW 2052 Australia; 2 Exchange student from Department of Materials and Metallurgical Engineering, Punjab Engineering College, Chandigarh (deemed to be University), Chandigarh, India; 3 Exchange student from Department of Mechanical Engineering, Punjab Engineering College, Chandigarh (deemed to be University), Chandigarh, India

## Abstract

When corrosion is the dominant failure factor in industrial application and at the same time high mechanical properties are required, aluminum bronze is one of the best candidates. Hence, there is a continuous quest for increasing the lifetime of aluminum bronze alloys through enhancing the abrasion and corrosion resistance. Existing methods are based on modifying the bulk properties of alloy or surface modification which required sophisticated equipment and process control. This approach has limited application for advanced components because of high price and difficulty to apply. In this research, we developed an innovative approach to enhance the corrosion and abrasion resistance of aluminum bronze through selective surface diffusion process. In this process, we have used waste materials as input and the modified surface has formed in a single and green process. New surface structure consists of finely dispersed kappa phase (χ ) in uniform alpha (α) solid solution matrix. Results have demonstrated that this uniform diffused modified surface layer has improved hardness of the base material and both corrosion and abrasion resistance has increased. This novel surface modification technique has opened a pathway for using waste materials as input for surface modification of aluminum bronze to meet the needs of industrial applications in a cost effective and environmentally friendly way.

## Introduction

Aluminium bronze 954 is a copper-based alloy, which has high corrosion and wear resistance along with high yield and tensile strength. Therefore, it is one of the most commonly used material in corrosion dominant applications such as marine industry^1,2^. Also, this alloy has used in landing gear parts of an air craft, pumps and ship building material for marine hardware^1^. Failure of material in these applications cause huge disasters hence require to be maintained and replaced from time to time, these replacement costs turn out to be a tremendous burden on the overall costs of the industry so there is a desperate need to devise a new and innovative method which increases the lifetime of the Al-bronze parts.

Several methods have been developed to enhance the properties of Al-bronze which is based on modifying the bulk properties or just modifying the surface properties. Some examples of these techniques are: (1) modification of microstructure by changing the bulk properties of the material i.e. by changing the microstructure through heat treatment, varying the temperature or changing the duration for which the metal is being heat treated^3^. (2) Surface modification using laser induced process and infrared thermography^4^ (3) Laser cladding Ni-Co duplex cladding in which Al_2_O_3_/Ni layers are inserted in between the copper substrate and laser cladding layers to avoid any defects in the laser cladding^5^.

These methods were successful, but they have their limitation, time consuming and not cost-effective. By increasing the hardness of bulk material, it will reduce the ductility^6^ of the whole structure which is not desirable in some cases. On the other hand, surface modification techniques which are using laser induced processes require very precise and complicated machinery in some cases involving infrared thermography^4^. In order to overcome these barriers, the current research suggested a diffusion of the elements similar to the alloying elements of the aluminium bronze such as Al and Fe using a single step *in situ* process and at the same time using waste as a source for providing these required elements.

Al in Al-bronze structure has two benefits, it forms a protective oxide layer on the surface of the alloy which increase the corrosion resistance of this material^1^ and at the same time its combination with Fe and Nickel (Ni) in the structure of alloy provides hardness^7^. For modifying the surface of this alloy using selective diffusion process we need a source of raw material which can provide these elements. By increasing the content of Al and Fe on the Al- bronze surface we will be able to increase its corrosion and abrasion resistance along with its hardness value.

Investigation into the constituents of steel slag revealed a high content of aluminum and iron in the form of oxides, which could be utilized for the diffusion process with prior reduction^6^ using waste plastics as a source of reductant. Slag is one of the most problematic waste in iron and steel making production lines in which has limited or no use and mainly destine in landfills or will be used as road base and other downgrading applications^8^. This complex waste stream consists of very useful and valuable elements which can be used as a resource for other applications. In the current study, steel making slag has been used as a source for providing Iron (Fe) and Aluminum (Al) for producing modified surface layer on Al-bronze alloy through a new innovative method.

This method is environmentally friendly as well as cost effective as it utilized the steelmaking slag and waste plastic as a resource. Our results demonstrate that a modified surface has been produced on the surface of Al-bronze which enhances the corrosion and abrasion resistance of the base structure. This novel method has a flexibility to achieve different properties by changing its parameter, can open a new approach for surface modification of Al-bronze alloy in a single step using waste as input.

## Experimental Procedure

Formation of a diffusion layer has been investigated using standard Al-bronze 954 with the chemical composition as mentioned in Table 1. Steel making slag with the chemical composition of Table 2 and mixed waste plastic has been used as input material for surface modification. For producing this new surface, Al-bronze samples were submerged in a mixture of steel making slag and mixed plastic in a graphite crucible and heat treated at 900 °C for 120 minutes^7^. Then the sample was quenched in water.

**Table 1:** Chemical composition of used aluminum bronze 954 (wt%) measured with spark spectroscopy.

Element	Aluminium (Al)	Iron (Fe)	Nickel (Ni)	Manganese (Mg)	Copper (Cu)
Weight Percentage	10–11.5	3.0–5.0	1.5	0.5	Balance

**Table 2:** Chemical composition of used steel making slag (wt%) measure with X-ray fluorescence (XRF).

Compound	Fe_2_O_3_	SiO_2_	MnO	CaO	Al_2_O_3_	MgO	Na_2_O	P_2_O_5_	Cr_2_O_3_
Weight percentage	38.6	12	7.2	30	4.3	7	0.3	0.4	2

After heat treatment samples were cut using a diamond cutter (Struers mini tomb) at speed 0.1 mm/sec to avoid generation of heat, then prepared by employing standard polishing techniques and etched in 5% Nital solution for about 25 minutes reveal its microstructure. Microstructure and thickness of this modified surface layer was examined with optical microscopy Nikon Eclipse ME600 and Hitachi S3400I scanning electron microscopy equipped with Energy-dispersive X-ray spectroscopy (EDS) analysis.

Mitutoyo Surftest SV-600 Profilometer was used to determine the surface roughness of the samples before wear testing and to scan the cross-sectional wear scar profile. Roughness is compared based on parameters such as R_a_ which is average surface roughness and R_q_ which is ten-point average surface roughness^10^. Wear resistance of Al-bronze samples were compared based on the wear performances which was studied by using Pin-on Disk technique according to the ASTM G99-05^10^. Wear rate is considered as a materials’ property which highly depends on the operating conditions. Therefore, the factors affecting wear behaviour of material were kept constant and samples were subjected to undergo 7960 laps (100 m) with a linear speed of 10 cm/s (roughly 500 laps/min) under constant load of 10 N. To maintain the similar roughness, all samples were polished using abrasive SiC paper and R_a_ and R_q_ were measured. Ethanol was used to clean the sample surface before roughness measurement and wear testing. A ruby ball having 6 mm diameter and 20 GPa hardness was used as a wear partner for all samples. To maintain the identical contact geometry, new ruby ball surface was selected for each wear run. The specific wear rate of the sample was determined using the following equation

1 $${\rm{SWR}}=\frac{{\rm{\Delta }}{\rm{w}}}{{\rm{\rho }}\times {\rm{L}}\times {\rm{D}}}$$

where the ${\rm{\Delta }}$w is the weight difference (before and after the test), which was determined using high precision weighing scale, ρ is the density of the materials which is same for all samples (8.8 g/cc), L is the applied load (10 N) and D is the sliding distance. Friction coefficient (${\rm{\mu }}$) was calculated using Equation 2 where F_f_ is the frictional force, which is captured via the load cell and L is the applied load

2 $${\rm{\mu }}=\frac{{{\rm{F}}}_{{\rm{f}}}}{{\rm{L}}}$$

The energy dissipated in the contact during the wear testing (Edesi) can be calculated using F_f_ and displacement in meter (ds) as the work of friction force as per Equation 3^11^. Energy dissipated at each interval was calculated and the total energy dissipated was obtained by adding all values.

3 $${{\rm{E}}}_{{\rm{desi}}}={\int }_{0}^{{\rm{\Delta }}{\rm{s}}}{{\rm{F}}}_{{\rm{f}}}{\rm{ds}}$$

The electrochemical properties of modified surface were considered by Tafel polarization test using Versatile Multipotentiostat VSP300 and obtained quantitative data was processed through EC-Lab® v11.10 software. The 3.5 wt% NaCl solution (pH = 6.4) was prepared as corrosive electrolyte for electrochemical measurement at room temperature 24 ± 1 °C. The potentiostat instrument was plugged to flat corrosion kit that consisted of three different channels to electrodes (Hg/HgCl_2_, saturated calomel electrode – SCE) for reference electrode, Pt foil as counter electrode and Al-bronze sample as working electrode. The open circuit potential (OCP) system at equilibrium state was monitored and recorded as corrosion potential value, *E*_corr_ after the immersion for 2 h of exposure to corrosive solution. Tested sample was delimited by an o-ring to achieve surface area of 1 cm^2^. Tafel polarisation curves were acquired between the range of −250 mV and 250 mV with respect to OCP at scanning speed of 0.5 mV/s. Determination of different corrosion current density values, *i*_corr_ can be defined by extrapolating linear section from each curve to *E*_corr_. Calculation of Tafel anodic (*b*_a_) and cathodic (*b*_c_) slopes (Δ*E*/log*i*) as the parts of the Tafel polarization plots can be determined.

## Results and Discussion

**Figure 1:**
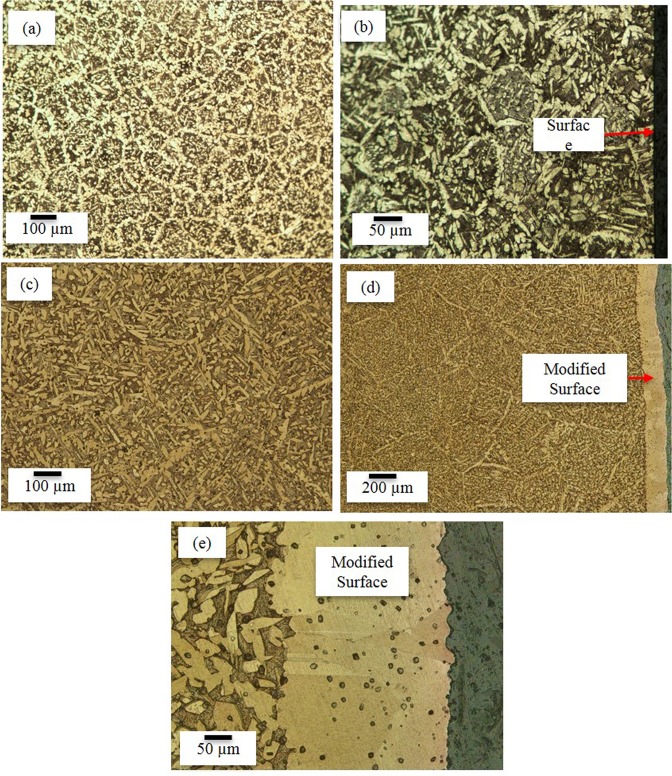
Characterization of Al-bronze using optical microscopy. (**a**) Optical microscopy showing the as received Al-bronze at the center. (**b**) Magnified image of as received Al-bronze at the edge of the sample. (**c**) Center of Al-bronze after selective diffusion heat treatment. (**d**) Image of the surface of heat treated sample. (**e**) Magnified structure of heat treated sample with modified surface.

**Figure 2:**
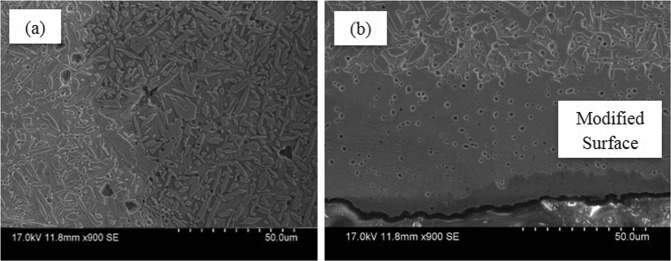
Characterization of Al-bronze using Hitachi s3400 I. (**a**) SEM imaging showing the microstructure at the center of Al-bronze after selective diffusion heat treatment, (**b**) SEM imaging showing the microstructure of surface of Al-bronze after selective diffusion heat treatment.

Optical microscopy of the Al-bronze sample showed a dual phase structure comprising α and β phases^3^ as shown in Fig. 1(a,b) for the samples before selective diffusion process. This structure is uniform in the sample from surface, Fig. 1b to center, Fig. 1a. After selective diffusion heat treatment using waste materials as a source of Fe and Al and diffusion of these elements into the structure Fe rich ϰ particles precipitate in a uniform α phase, Fig. 1d,e and Fig. 2a,b. The center of the structure, Fig. 1c and Fig. 2a consists of α and β phase with some dispersed ϰ phase which formed due to heat treatment at 900 °C for 120 minutes and quenching in the water.

This newly formed modified surface has 200 µm uniform thickness, Fig. 1d and Fig. 2b. In its bulk region of modified surface Al-bronze alloy, the iron rich ϰ phase precipitated out of β and α phases resulting in reduced β phase in the microstructure whereas volume fraction of β phase remained similar but decreased in iron content leading to rich in Al, Fig. 1c. The brighter colored elongated phase is α (red) and darker colored, globular shape structure is ϰ (blue) which is an intermetallic of Al-Fe and needle shaped structure having sharp edges visible in the background of the α is the β (yellow) martensite^7^.

The modified surface structure consists of uniformly dispersed fine ϰ phase which is hard and brittle and rich in Fe in ductile α^12^ phase which is rich in Al^7,13^. Previous study has proven that by heat treatment of waste plastics at high temperature, these polymers degrade and producing CO, CO_2_ and CH_4_^14^ and solid carbon. Generated gases and solid carbon will reduce the Fe_2_O_3_ and Al_2_O_3_ in steel slag and reduced it to form free Fe and Al atoms on the surface of Al-bronze. This Fe and Al atoms will diffuse into the structure and forms the α and the ϰ^13^ structure on surface.

Furthermore, the β martensite was developed when the sample was quenched in water, which increased the hardness because of the high lattice distortion^7,13^. The scanning electron microscopy of the sample shows the elongated β martensite and globular ϰ dispersed in uniform α phase.

### Hardness

The hardness test was done on the Struers machine proved that the hardness of the samples before and after formation of modified surface. Hardness Vickers values comparison before and after surface modification of Al-bronze alloy have been summarised in Table 3. Hardness of Al-bronze sample has increased to 270 1HV from 228.6 1HV. This increase in the hardness is the direct effect of formation of uniform α phase which contains fine and dispersed ϰ phase as it is visible in Fig. 1c.

**Table 3:** Hardness of sample before and after formation of modified surface.

	Before	After
Hardness	228.67	270.21
Std. Deviations	2.08	7.94

### Abrasion

**Figure 3:**
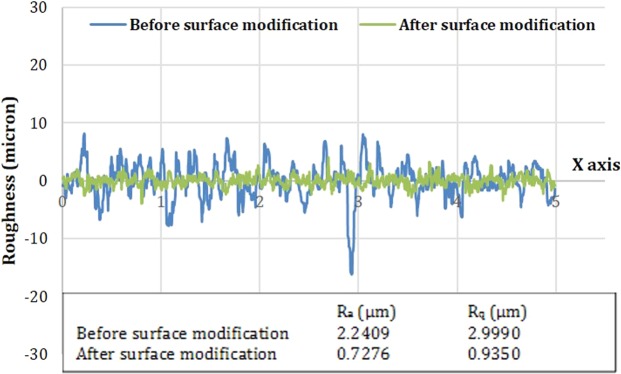
Surface roughness of Al-bronze sample before and after surface modification.

Figure 3 shows the change in the roughness of the Al-bronze samples after surface modification. The numbers in inset represent the average surface roughness showing the overall description of height variations (R_a_), and deviation from main line (R_q_).

**Figure 4:**
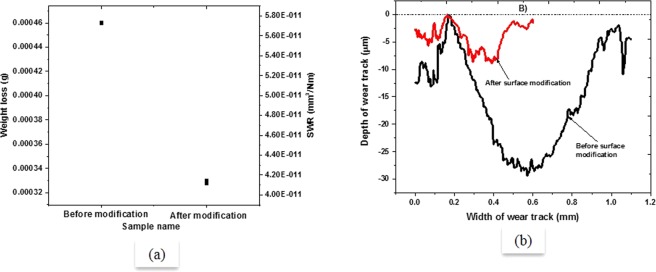
Weight loss and specific wear rate of Al-bronze sample before and after surface modification.

**Figure 5:**
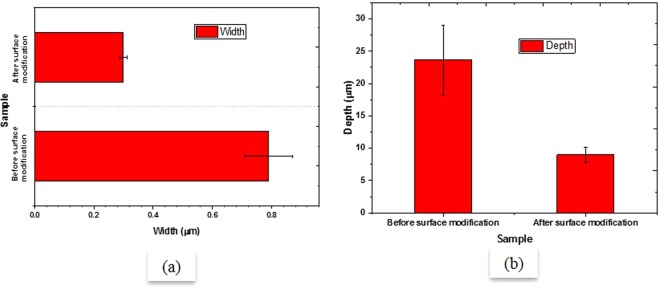
Change in depth and width of wear scar after modification of Al-bronze sample.

Figure 4(a) shows the comparison of weight loss in g of Al-bronze samples before and after surface modification on primary axis and SWR on secondary axis. The weight loss of the sample before surface modification was found to be higher as compared to that after surface modification. In other words, modified sample showed improved wear resistance as compared to unmodified sample. The increase in wear resistance of Al-bronze sample can relate to higher hardness of the newly formed layer after modification which predominantly has ϰ phase embedded in the softer α phase with the α phase in abundance which prevented the ϰ phase to be peeled off from the structure being brittle and hard. The study of wear scar (shown in Fig. 4(b)) showed that the depth of wear scar was completely in the modified layer (i.e. roughly 100–150 µm) which resulted into increased wear resistance due to presence of hard ϰ phase having intermetallic Fe_3_Al (as observed in previous study). As can be observed in Fig. 5 (also can be observed in Fig. 4(b)), width and depth of wear track of Al-bronze samples were also decreased comprehensively after modification of the surface.

**Figure 6:**
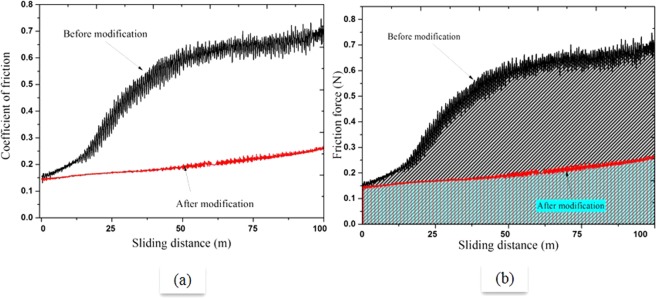
Comparison of COF and friction force of Al-bronze sample before and after surface modification.

The friction coefficient of Al-bronze samples against the sliding distance of 100 m under load of 10 N is displayed in Fig. 6(a). The initial region of the graph represents the sliding wear mechanism, (also mentioned as running-in-stage in the literature^15^), shows different behaviour of both samples. In case of the sample before modification, coefficient of friction (COF) was increasing rapidly which shows the higher friction between the samples and wear partner. After few laps, COF attends steady state due to the presence of wear debris generated in the running-in-stage, which act as third body in wear system. Once the system transforms to abrasive wear from sliding wear, the nature of friction depends on the properties of debris such as hardness. On the other hand, Al-bronze sample after surface modification showed low friction between samples surface and wear partner in order to attend the relative speed of the wear partners. Morphological surface of as received Al-bronze sample exhibited two phases (see Fig. 1a,b), which possibly resulted into small variation into friction or COF that can be seen in Fig. 6 in the form of noise. Whereas sample surface after modification contains predominantly one phase (FeAl) hence showed less variation in friction. Table 4 shows mean COF of modified and unmodified Al-bronze samples after 100 m wear. Figure 6(b) shows the energy dissipated during the friction of ruby ball and Al-bronze samples before and after modification. The energy dissipated in 100 m wear of Al-bronze sample after modification was 56% lower as compared to that of before modification.

**Table 4:** Mean COF of Al-bronze samples during wear against ruby for 100 m and energy dissipated in the same.

Al-bronze Sample	Mean COF	Energy dissipated (Joules)
Before modification	0.52 (±0.16)	52.32
After modification	0.23 (±0.08)	22.93

### Corrosion

During the corrosion of Al-bronze sample the β martensitic phase acts as an anode therefore, preferential corrosion attack occurs on β martensitic^16^. However, in modified surface Al-bronze sample, a continuous α phase with finely dispersed ϰ had been generated. As a result, the corrosion resistance increases as there is a continuous microstructure which has higher corrosion resistance.

**Figure 7:**
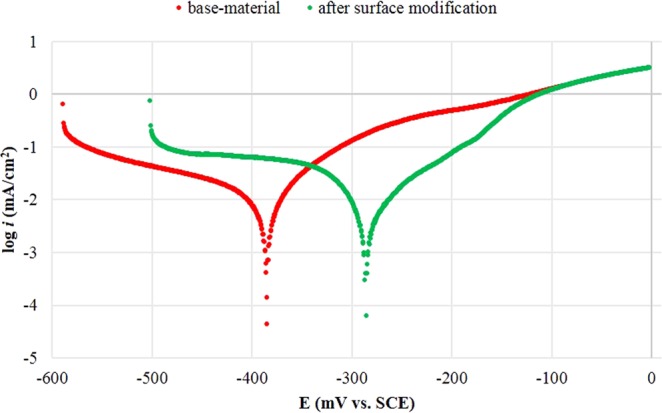
Tafel polarization curves on base-sample and modified surface of Al-bronze samples after immersion in 3.5 wt% NaCl solution for 2 h.

Electrochemical corrosion test in 3.5 wt% NaCl electrolyte solution was performed to obtain Tafel polarization curve that provides valuable information to predict different corrosion rate. From the intersection of cathodic slope (left section – during charging) that exhibited the H_2_ transformation reaction and anodic slope (right section – during discharging) that presented the metal dissolution chemical reaction, the *i*_corr_ values can be defined through extrapolation of cathodic branch line up to a spot where vertically it crosses the *E*_corr_^17,18^. In Fig. 7, the Tafel extrapolation plots were generated that by applying surface modification decreased the corrosion rate.

Copper, aluminum and ferrous along with nickel in the aluminium bronze form oxide layers on reaction with atmospheric oxygen which bonds with the base Al-bronze and shows good resistance to fluid flow velocity corrosion, alumina layer of aluminium oxide being harder provides good resistance to erosion and corrosion^16,19^.

The corrosion parameters measured from Tafel curves for base-material and after modification of Al-bronze samples are presented in Table 5 commonly with a higher value of E_corr_ and lower level of i_corr_ showed improvement in corrosion resistance properties. Moreover, to measure the corrosion protection of modified surface, the values of *R*_p_ were calculated from Tafel constants anodic, *b*_a_ and cathodic, *b*_c_ values through Stern-Geary equation^20^, as follows:

4 $${R}_{{\rm{p}}}=\frac{{{\rm{b}}}_{{\rm{a}}}\ast {{\rm{b}}}_{{\rm{c}}}}{2.303\ast ({{\rm{b}}}_{{\rm{a}}}+{{\rm{b}}}_{{\rm{c}}})}\ast {{\rm{i}}}_{{\rm{corr}}}$$

After *R*_p_ values have been defined, the efficiency of corrosion protection from modified surface (*η*_*EF*_) in percentage unit can be calculated against the base-material Al-bronze according to the expression (5)^21^:

5 $${\eta }_{EF}=100\ast \frac{{{\rm{R}}}_{{\rm{p}}}^{-1}({\rm{uncoated}})-{{\rm{R}}}_{{\rm{p}}}^{-1}({\rm{coated}})}{{{\rm{R}}}_{{\rm{p}}}^{-1}({\rm{coated}})}$$

Both results in Fig. 7 and Table 5 demonstrated that the higher value of *E*_corr_ and lower value of *i*_corr_ were the Al-bronze sample after the surface modification to the Al-bronze substrate. Additionally, *E*_corr_ value of sample after surface modification contributed of 12.33% more toward the noble side than base-material. This innovative coating from waste-based input had performed a substantial increase in its protection efficiency, *η*_EF_ for up to 13.83% after surface modification applied to the base-material sample. Therefore, with the presence of diffused surface modification was found to be perform as the protective barrier for anti-corrosive properties resulted from increased *E*_corr_ with decreased in i_corr_ values.

**Table 5:** Tafel polarization parameters before and after surface modification of Al-bronze sample immersed in 3.5 wt% NaCl solution.

Sample-ID	Electrochemical Corrosion Measurements					
	*E*_corr_ (mV vs. SCE)	*i*_corr_ (mA/cm^2^)	*b*_a_ (mV/dec)	*b*_c_ (mV/dec)	*R*_p_ (kΩ.cm^2^)	*η*_*EF*_ (%)
base material	−385	−1.86	77.4	74.3	45.11	—
after surface modification	−284	−2.26	200.5	189.1	52.35	13.83

## Conclusion

Identifying the best practice for modifying the surface of Al-bronze alloy in a cost-effective process is in high demand for industrial application. In this study, a modified surface on Al-bronze alloy has been successfully produced through selective diffusion process using steel making slag and mixed waste plastic as a source of input materials. Modified surface layer consists of fine dispersed hard kappa phase (ϰ) in relatively softer alpha phase (α) solid solution matrix which makes it ideal structure for abrasion resistance. This newly formed surface has increased the hardness of Al-bronze by 17% and at the same time drastically increased the abrasion resistance. It also acted as a barrier to reduce susceptibility to corrosion with evidence of more positive on corrosion potential value to noble side and lower current density value against the base-material of Al-bronze – *η*_*EF*_ accounted to 13.83%. Benefits of this new approach for modifying the surface of Al-bronze are twofold. It has formed a uniform modified surface which improved both corrosion and abrasion resistance of base material and at the same time it is simple one step process which has used waste materials as input.

## Data Availability

The data that support the findings of this study are available from the corresponding author upon reasonable request.
